# The Contribution of Neutrophil Extracellular Traps to Coagulopathy in Patients with COVID-19-Related Thrombosis

**DOI:** 10.3390/v16111677

**Published:** 2024-10-27

**Authors:** Carolyn Enochs, Gabriela Delevati Colpo, Lucy Couture, Lynae Baskin, Ana E. Cahuiche, Eunyoung Angela Lee, Shahid Nimjee, Louise D. McCullough

**Affiliations:** 1 Department of Neurology, University of Texas Health Science Center at Houston, McGovern Medical School, Houston, TX 77030, USA; carolynenochs01@gmail.com (C.E.); gabriela.d.colpo@uth.tmc.edu (G.D.C.); lynae.baskin@uth.tmc.edu (L.B.); ana.e.cahuichesaiz@uth.tmc.edu (A.E.C.); eunyoung.lee@uth.tmc.edu (E.A.L.); louise.d.mccullough@uth.tmc.edu (L.D.M.); 2 Neurosurgery, The Ohio State University Medical Center, Columbus, OH 43210, USA; shahid.nimjee@osumc.edu

**Keywords:** coronavirus, COVID-19, thrombosis, viral infection, epidemiology, coagulation, neutrophil extracellular trap

## Abstract

COVID-19 is caused by severe acute respiratory syndrome coronavirus 2 (SARS-CoV-2) and is associated with hypercoagulability and increased incidence of thrombotic events. In this study, we investigated the levels of neutrophil extracellular trap biomarkers and von Willebrand factor to assess if these could predict the occurrence of a thrombotic event in COVID-19 patients. We enrolled 202 patients hospitalized with symptomatic COVID-19 infection. Of those, 104 patients did not experience any type of thrombotic events before or during their hospitalization. These patients were compared to the other cohort of 98, who experienced thrombotic events before or during their hospitalization. In total, 61 patients who experienced thrombotic events had the event after initial blood collection, so the predictive capacity of biomarkers in these patients was evaluated. Citrullinated histone H3 was the best predictive biomarker for thrombotic events in COVID-19 regardless of age, sex, and race; disease severity was also a significant predictor in most thrombotic event groups. These results may better inform treatment and prophylaxis of thrombotic events in COVID-19 and similar viral illnesses in the future to improve outcomes and reduce mortality.

## 1. Introduction

COVID-19 is caused by the highly contagious severe acute respiratory syndrome coronavirus 2 (SARS-CoV-2). It commonly manifests with influenza-like symptoms and viral pneumonia, progressing in severe cases to acute respiratory distress syndrome (ARDS) and multi-organ failure [1]. COVID-19 infection, similar to other respiratory infections, is associated with an elevated risk of hypercoagulability, leading to an increased incidence of thrombotic events [2,3,4,5]. Higher levels of D-dimer and fibrinogen are seen in the blood of patients hospitalized for COVID-19, which have been linked to a more severe disease course and poorer outcome [6,7]. COVID-19 coagulopathy is characterized by thrombotic events, on both the micro- and macrovascular scale, and it is a life-threatening complication of severe SARS-CoV-2 infection [8].

The innate immune response is the first line of defense against pathogens, mediated in large part through the activation of neutrophils, the most abundant population of circulating leukocytes [9,10,11]. Neutrophils phagocytose pathogens and expose them to intracellular bactericidal compounds. Neutrophils also produce neutrophil extracellular traps (NETs), net-like structures that help trap and kill pathogens outside of the cell [12]. NETs are formed by chromatin meshes, antimicrobial peptides, and enzymes that, when released into the extracellular space, immobilize microorganisms and facilitate their death [13,14,15]. NETs have been implicated in a process called thromboinflammation, which has been shown to be a major pathway activated in COVID-19 and is also associated with ischemic stroke [16]. NETs are also an essential component contributing to the multi-organ complications of COVID-19, including immunothrombosis [17]. In addition, NETs activate coagulation pathways that lead to thrombus formation and play a role in inflammation but are also an important mechanism used to reduce the spread of pathogens [18].

Neutrophil elastase (NE), a component of NETs, can directly modify platelet function to enhance fibrin formation by activating platelets through protease-activated receptor 4 (PAR4) and thrombin [19,20]. Another component of NETs, the peroxidase enzyme myeloperoxidase (MPO) is primarily secreted by neutrophils and is a major scavenger of the pro-oxidant hydrogen peroxide [21]. MPO has been found to be significantly elevated in the plasma of COVID-19 patients compared to healthy controls [17]. The most abundant content found in NETs are nuclear components and histones. Extracellular histones, such as citrullinated Histone H3 (CitH3), exert direct cytotoxic effects through membrane disruption, increased intracellular calcium, activation of Toll-like receptors, and inflammasome and complement activation [22,23].

Von Willebrand factor (vWF), synthesized primarily in endothelial cells, is rapidly released into the bloodstream and mediates platelet adhesion to damaged blood vessels facilitating thrombus formation [24,25,26]. VWF interacts directly with the DNA of NETs, promoting thrombus formation in the setting of tissue damage or inflammation [27]. Whether these pathways are specifically activated in patients with COVID-19-related thrombosis is unclear. As increased vWF levels are a reliable predictor of future venous and arterial thromboembolic events, levels of vWF and NETs could serve as potential biomarkers of risk for COVID-19-related thrombosis and allow for improved monitoring of future thrombotic event risk [28,29].

In this study, we evaluated NET markers and vWF levels in the plasma of COVID-19 patients, with or without a thrombotic event during hospitalization, to identify if these biomarkers are associated with an increased risk of thrombosis in patients with COVID-19 and can predict thrombotic events in COVID-19 patients.

## 2. Materials and Methods

### 2.1. Study Population and Patient Inclusion and Exclusion Criteria

This study was conducted at the Memorial Hermann Hospital System and UTHealth in Houston, TX, USA. Patients were enrolled at three different hospital sites, all with diverse patient demographics. We prospectively enrolled 202 patients hospitalized with acute symptomatic COVID-19. Patients were enrolled between September 2020 and January 2022. Among these 202 patients, 104 patients were classified as ‘non-thrombotic event COVID-19’ patients. This cohort of patients did not experience any thrombotic events throughout their hospitalization. The remainder of the patient cohort (n = 98) presented with acute COVID-19 and a thrombotic event immediately prior to or during the relevant hospitalization with diagnoses including ischemic stroke, deep vein thrombosis, pulmonary embolism, cerebral venous thrombosis, or myocardial infarction. Out of these 98 patients, 37 of them had a chief complaint related to their thrombotic event at admission, while the remaining 61 patients were admitted for COVID-19 treatment and suffered a thrombotic event while in the hospital. Blood was obtained prior to the thrombotic event in these 61 cases. Five of these 61 patients were excluded due to other factors that could contribute to coagulopathy, including pregnancy (n = 1), history of chronic thrombotic events (n = 1), recent history of dialysis treatment (n = 1), and traumatic hemorrhage as reason for their hospitalization (n = 2). Therefore, a total of 56 patients who had a thrombotic event after blood was obtained were included. Thrombotic events were determined by a review of patients’ electronic medical records (EMRs) and categorized utilizing the patient’s final International Classification of Disease, Tenth Revision (ICD-10) codes upon their discharge. ICD-10 codes were extracted from patients’ charts to categorize them into the thrombotic event arm of this study.

Inclusion criteria for hospitalized patients were laboratory-confirmed SARS-CoV-2 infection by real-time polymerase chain reaction, written/verbal informed consent from the patient or surrogate, and age ≥ 18 years. Exclusion criteria for hospitalized COVID-19 patients are as follows: recent blood transfusions, chronic steroid or immunosuppressant use, history of chronic thrombosis, pregnancy, and active dialysis treatment.

### 2.2. Samples

Peripheral blood was collected in sterile vacutainers within the first 72 h after admission and transported on ice for immediate processing. Plasma was isolated by centrifuging samples at 1200× *g* for 10 min at 4 °C, followed by plasma supernatant isolation and further centrifugation at 10,000× *g* for 10 min at 4 °C to generate plasma samples. Samples were then aliquoted and stored at −80 °C until analysis.

### 2.3. Biochemical Analysis

Plasma samples were used to quantify the levels of vWF (MBS704140), citrullinated histone H3 (MBS1609017), neutrophil elastase (MBS9425309) and myeloperoxidase (MBS2886648) by Enzyme-Linked Immunosorbent Assay (ELISA) kits according to the procedures supplied by the manufacturer (MyBioSource, San Diego, CA, USA). All assays were performed by individuals blinded to COVID-19 diagnosis.

### 2.4. EMR Data Analysis

ICD-10 codes were extracted from patient charts to categorize thrombotic event occurrence, including I.63 (cerebral infarction), I.81 (portal vein thrombosis), I.82 (other venous embolism and thrombosis), I.74 (arterial embolism and thrombosis), I.21 (acute myocardial infarction), I.22 (ST elevation (STEMI) myocardial infarction and Non-ST elevation (NSTEMI) myocardial infarction), I.24.0 (coronary thrombosis not resulting in myocardial infarction), and I.26 (pulmonary embolism). Of the 98 patients who experienced a thrombotic event, 26 of those clots were from a venous source while the remainder (n = 72) were determined to be due to arterial thrombi. The patient’s oxygen requirement and past medical history were collected from hospital charts. Additional patient laboratory data were collected from the electronic medical record, including levels of D-dimer, fibrinogen, segmented neutrophil count, total neutrophil count, Protime, aPTT, and INR from the most complete labs drawn closest to the date and time our blood sample was collected.

All patients received the standard-of-care prophylaxis treatment, which included heparin or enoxaparin. This was confirmed by chart review after discharge.

### 2.5. Statistical Analysis

Demographic variables, including age, sex, race, and severity, as well as the levels of biomarkers between the groups, are compared by the Chi-square test for categorical variables and the Wilcoxon rank-sum test for continuous variables. Univariable logistics regression analysis was conducted to find the risk factors associated with thrombotic events in COVID-19 patients. The significant risk factors less than 0.1 from the result of the univariable logistic regression analysis were included in the multivariable logistic regression analysis. Subgroup analysis was also conducted to examine the risk of thrombotic events at different levels of potential confounding factors such as race, sex, and age group. Receiver-Operating Characteristic (ROC) Analysis was performed to evaluate predictive models with identified risk factors to classify patients with thrombotic events. The area under the curve (AUC) was provided with 95% confidence intervals and compared. Additionally, Spearman’s rho correlation was computed and plotted in heatmaps to examine if there is any relationship between NET biomarkers and coagulation factors for each cohort. The statistical analyses were performed using R statistical software (R 4.3.1, R Foundation for Statistical Computing, Vienna, Austria) and GraphPad Prisma 8.4.3. Two-tailed *p* < 0.05 was considered significant.

## 3. Results

The demographic and clinical characteristics of the study participants are shown in Table 1. The two groups (COVID-19 without thrombosis and COVID-19 with thrombosis) were comparable in age, sex, and race distribution. To analyze the selected biomarkers across the patient cohorts, we investigated levels between groups as well as their capacity to predict thrombotic events. We used only the 56 patients who had a thrombotic event post-blood collection in this analysis so that we could assess whether any of the biomarkers could predict a thrombotic event in COVID-19. Of the 56 patients used in the predictive analysis, 19 patients had venous thrombosis and the remaining 37 had arterial thrombosis. Of the 19 patients diagnosed with venous thrombotic events, nine patients experienced a left laterality thrombosis, nine experienced right-sided thrombosis, with one patient with unspecified laterality. With respect to the location of thrombosis for these patients, 12 patients experienced thrombosis within their lower extremities, with the remaining seven patients experiencing upper extremity thrombosis. For patients who experienced an arterial thrombotic event (n = 37), 18 patients experienced a myocardial infarction, with the remaining 19 patients experiencing different varying types of thrombotic events such as ischemic strokes with etiologies of cardioembolic, intracranial atherosclerotic disease (ICAD), or large artery atherosclerosis. There was a 31.58% mortality rate (n = 6) associated with venous thrombosis, whereas there was a 29.73% mortality rate (n = 11) for those who experienced an arterial thrombotic event during their hospitalization.

First, we compared NET markers. CitH3 was significantly higher in COVID-19 patients with thrombotic events when compared to COVID-19 patients without a thrombotic event (*p* < 0.001) (Table 1). Neutrophil count was also significantly higher in the thrombotic event group compared to the non-thrombotic event group (*p* = 0.031) (Table 1).

We then analyzed the biomarker levels across COVID-19 severity. Severity was categorized according to the NIH guidelines into asymptomatic, mild, moderate, severe, and critical (COVID-19 Treatment Guidelines Panel. Coronavirus Disease 2019 (COVID-19) Treatment Guidelines). There were no differences in the biomarker levels across COVID-19 severity. We then separated the entire patient population into higher-severity and lower-severity groups, with the higher-severity group including severe and critical patients and the lower-severity group including mild and moderate patients. Mild and moderate patients were those hospitalized with various signs and symptoms of COVID-19, including shortness of breath, fever, malaise, and/or lower respiratory disease during clinical assessment, but with oxygen saturation (SpO_2_) >94% on room air at sea level and who did receive oxygen support. Patients in the severe and critical classification included those with a saturation of oxygen <94% on room air at sea level, lung infiltrates >50%, or respiratory failure, septic shock, and/or multiorgan dysfunction. There were significantly more lower-severity patients in the no-thrombotic event group compared to the thrombotic event group (*p* < 0.001) (Table 1). This is important to note that while all critical patients required mechanical ventilation, the patients in the severe classifications were on high-flow devices (Vapotherm, BIPAP, Optiflow), there were no significant differences in levels based on separate classifications, and only when combined into larger groups (high-severity and low-severity groups) was there a significant difference.

Both univariable and multivariable logistic regression analyses were performed to determine the ability of the biomarkers to predict a thrombotic event. The significant risk factors for a thrombotic event were found to be COVID-19 severity, citH3 levels, and neutrophil count (*p* < 0.0001 for severity for both univariable and multivariable, *p* = 0.0159 and *p* = 0.0302 for citH3 for univariable and multivariable, and *p* = 0.0257 and *p* = 0.0508 for neutrophil count for univariable and multivariable) (Table 2). The odds of having a thrombotic event were 6.17 times higher in the higher-severity group than the lower-severity group according to the univariable analysis, and 4.84 times higher in the multivariable analysis (Table 2). Age, sex, and race were not found to be significant factors in thrombotic event occurrence. Some of the EMR-derived variables such as D-dimer, Protime, aPTT, INR, and fibrinogen were omitted from the analysis due to high levels of missingness.

We also analyzed the predictive capacity of the biomarkers across race. We defined the groups as White, Black, Other, and Asian. The White cohort includes people identifying as White Non-Hispanic and White Hispanic, and the majority of the patients in the “Other” cohort represented Hispanic and Latino populations. The Asian population was too small in comparison to our other racial groups, so they were excluded in the analysis (n = 6). We determined the patients’ race based on self-reporting from the electronic medical record or health history survey collection.

We found that severity was a significant predictor of having a thrombotic event regardless of race, corroborating our earlier findings (*p* = 0.0295 in Black, *p* = 0.0042 in White, and *p* = 0.0224 in Other) (Table 3). Interestingly, in the White population, vWF, citH3, and neutrophil count were also found to be significant predictors of thrombotic events (*p* = 0.0505, *p* = 0.0105, *p* = 0.0277, respectively) (Table 3). These were not found to be significant in the other racial groups, Black and Other, even though citH3 and neutrophil count were significantly higher when comparing the overall thrombotic event group to the non-thrombotic event group.

When analyzing the multivariable logistic regression by sex, severity was significant regardless of sex (*p* = 0.0012 in males and *p* = 0.0062 in females) (Table 3). VWF and citH3 were also significant predictors of thrombotic events in all COVID-19 hospitalized females (*p* = 0.0305 and *p* = 0.0206, respectively) (Table 3).

We also determined if the predictive biomarkers changed based on age. We separated all COVID-19 hospitalized patients into age less than 60 and age greater than or equal to 60. Severity was a significant predictor in both groups (*p* = 0.0500 in age < 60, *p* = 0.0008 in age ≥ 60) (Table 3). Interestingly, in the age group less than 60, citH3 and neutrophil count were found to be significant predictors of thrombotic event occurrence (*p* = 0.0343 and *p* = 0.0271, respectively), but were not significant predictors in the age group above 60 (Table 3).

Utilizing a classification model and ROC curve, we analyzed if our significant prediction factors had good classification ability to determine if our chosen factors are strong predictors of a thrombotic event occurring in hospitalized COVID-19 patients. Combining severity, vWF, citH3, and neutrophil count into one model, the 95% confidence interval indicated fair classification performance, demonstrating that these variables can significantly predict a thrombotic event (AUC = 0.7795, [0.6962, 0.8628]) (Table 4). When separated into models based on each variable, severity (AUC = 0.7004, [0.6229, 0.7778], *p* < 0.0001), citH3 (AUC = 0.7087, [0.6203,0.7971], *p* < 0.0001), and neutrophil count (AUC = 0.5999, [0.5031, 0.6968], *p* = 0.0432) had significant classification performance (Table 4). The higher AUC for citH3 demonstrates that this NET marker has the best ability to predict a thrombotic event compared to the other variables tested. This verifies our above findings that COVID-19 patients experiencing thrombotic events have higher levels of citH3.

**Figure 1:**
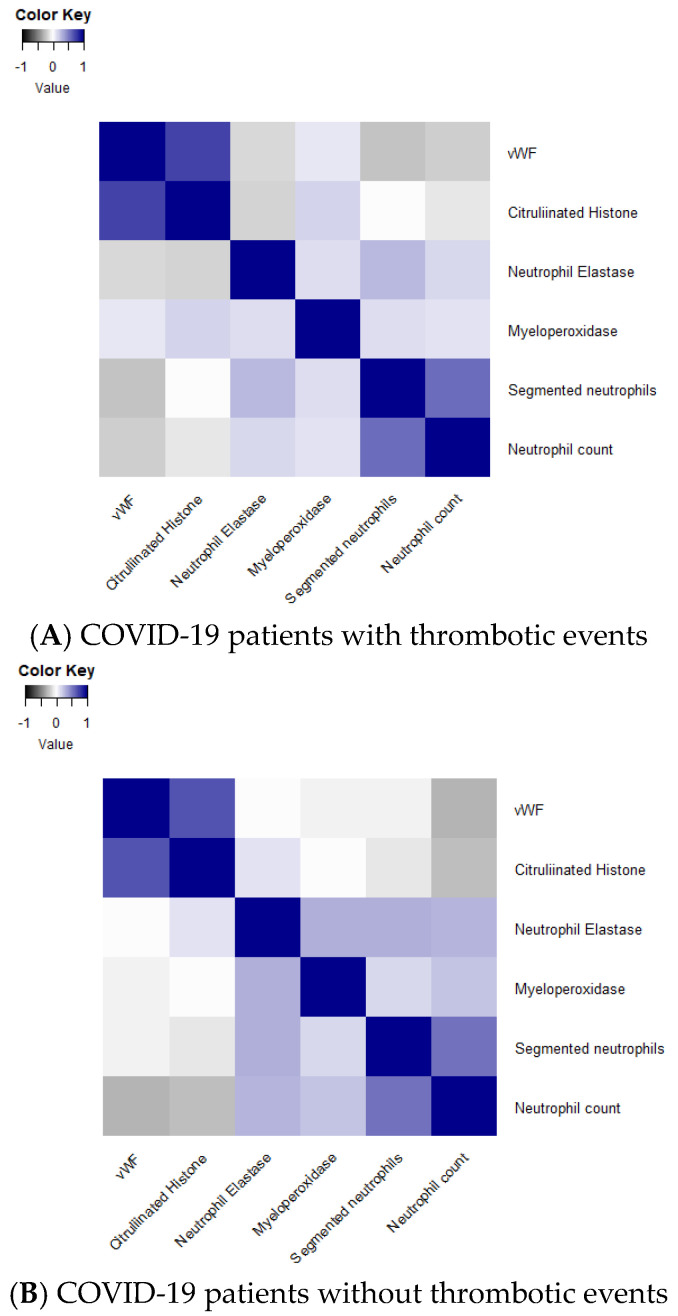
Heatmap of Spearman’s Rho correlation in COVID-19 patients with and without thrombotic events. (**A**) is a heatmap representing the thrombotic event group from Table 5; (**B**) is a heatmap representing the no-thrombotic event group from Table 5.

Then, a Spearman’s rho correlation was performed to assess whether individual NET biomarkers were correlated with each other and with coagulation factors in all COVID-19 patients. VWF and citH3 were found to be highly correlated in patients with a thrombotic event, demonstrating that as citH3 increases, vWF also increases (ρ = 0.75 and *p* < 0.0001) (Table 5, Figure 1A). There was also a significant correlation between neutrophil count and segmented neutrophils in patients with thrombotic events (ρ = 0.59 and *p* < 0.0001) (Table 5, Figure 1A). In patients without a thrombotic event, there was a significant correlation between vWF and citH3 and between vWF and neutrophil count (ρ = 0.67, *p* < 0.0001 and ρ = −0.29, *p* = 0.0038, respectively) (Table 5, Figure 1B). There was also a significant correlation between citH3 and neutrophil count (ρ = −0.22, *p* = 0.0269) (Table 5, Figure 1B). Neutrophil elastase was found to be significantly correlated with myeloperoxidase, segmented neutrophils, and neutrophil count (ρ = 0.32, *p* = 0.0016; ρ =0.30, *p* = 0.0030; ρ = 0.29, *p* = 0.0037, respectively) (Table 5, Figure 1B). Myeloperoxidase and neutrophil count were also significantly correlated (ρ = 0.24, *p* = 0.0217) (Table 5, Figure 1B). Lastly, segmented neutrophil count and neutrophil count were significantly correlated (ρ = 0.57, *p* < 0.0001) (Table 5, Figure 1B).

## 4. Discussion

Inflammation and hypercoagulability have been extensively studied since the surge in COVID-19. Several studies have measured NET markers in the blood of patients with COVID-19, and overall, our results corroborated previous findings that COVID-19 patients have significantly elevated levels of NETs, including MPO and citH3 [30,31]. It has also been demonstrated that these NET levels are higher in more severe patients and in patients who experience thrombosis during COVID-19 [32]. However, our study is one of the largest investigating NET biomarkers within COVID-19 patients during hospitalization, and one of the only to look at risk factors that correlate to thrombotic events.

The hypercoagulable state that many experience during COVID-19 illness has been suggested to occur as a result of the activation of the body’s natural coagulation cascade due to the endothelial damage that occurs with this virus-associated endotheliopathy [33,34,35]. When the coagulation cascade is activated, thrombin generation occurs and blood clots form. When inflammation occurs, further coagulation occurs, exacerbating this issue. Much research has occurred determining that thromboembolic events are a common occurrence in COVID-19 illness due to this hypercoagulability [36]. This activation of the coagulation cascade can lead to the exacerbated activation of cytokines, colloquially known as a ‘cytokine storm’ [37]. This influx of proteins can lead to increased severity in hospitalized COVID-19 patients, associated with high systemic levels of IL-6 β, TNF-α, and IL-6 [38]. It is commonly associated that interleukin cytokines, especially IL-6 β, IL-6, and IL-8, are involved in hyperacute inflammation.

Coagulopathy in COVID-19 causes patients to experience an increase in thrombotic complications, which may be due to a higher risk of venous and arterial thromboembolic complications possibly induced by the immunothrombotic state [39]. Interestingly, even COVID-19 patients not admitted to critical care units showed a higher incidence of thromboembolic complications compared to community-acquired pneumonia patients not admitted to critical care units [40]. Multiple prior studies have reported high rates of thromboembolism in both critically ill and non-critically ill hospitalized COVID-19 patients, despite routine thromboprophylaxis therapy [41]. However, one meta-analysis found that in ventilated COVID-19 patients, there was a lower mortality rate for those treated with anticoagulation [42].

Thrombotic events are one of the leading causes of morbidity and mortality in hospitalized COVID-19 patients [43]. One study found that COVID-19 patients who experienced a thrombotic event were more likely to be in the ICU than COVID-19 patients who did not experience a thrombotic event, suggesting that severity plays a role, which was also found in our cohort [40]. This study also found that the occurrence of a thrombotic event with COVID-19 was associated with an approximately five times higher risk of death than patients with community-acquired pneumonia. The ability to determine who may be at the highest risk for thrombotic event occurrence would assist physicians to help guide preventative strategies. Our study examined the interaction between biomarkers and disease severity on thrombosis, with none of the biomarkers returning significant results. These results indicate that the effect of severity does not depend on the level of biomarkers present.

Many clinical trials at the beginning of the pandemic investigated the use of prophylactic anticoagulation therapy in hospitalized COVID-19 patients. However, there are still many unknowns from this data, as many studies terminated early due to low enrollment once case numbers decreased. Most of these studies also lacked data informed by specific biomarkers. Gu, et al. found that the prophylactic use of anticoagulants during hospitalization in COVID-19 patients was effective in patients with pre-existing cardiovascular risk factors or disease [43]. McQuilten, et al. found an 86% probability that intermediate-dose thromboprophylaxis reduced mortality by day 28 of COVID-19 in hospitalized, predominantly South Asian patients [41]. In the PREVENT-HD trial, Piazza, et al. concluded that the anticoagulant rivaroxaban did not significantly decrease the frequency of thromboembolic events in non-hospitalized COVID-19 patients; however, this trial was stopped prematurely due to recruitment challenges [44]. In another related study in Germany, COVID-PREVENT, Rauch-Kröhnert reported similar results but from hospitalized COVID-19 patients, suggesting no benefit for rivaroxaban [45]. However, the author did not use any biomarker levels besides D-dimer. Wang, et al. similarly assessed the effect of using anticoagulation to decrease mortality due to thromboembolic events in non-hospitalized COVID-19 patients [46]. They had similar challenges to Piazza, et al. and had to conclude the study prematurely, resulting in inconsistent data.

The role of race in COVID-19-related outcomes has been previously examined. African American populations have worse outcomes when compared to White or Asian populations [47]. Interestingly, African Americans had higher levels of vWF compared to White and Hispanic individuals [47]. However, we found that vWF, citH3, and neutrophil count were only significant predictors of thrombosis in COVID-19 in our White population; however, these results need to be confirmed in a larger population since our sample size is small when divided by race. Therefore, our data do not elucidate any biomarker that would be more beneficial to use for screening of thrombotic risk in Black individuals, despite their higher risk.

Additionally, prior studies have shown that the risk of thrombosis is greater in males than females [42,48]. Studies have also demonstrated that males are at greater risk of mortality, especially in younger age groups, from COVID-19 [49]. Similar to our findings on the lack of effective predictive outcomes for race, our predictive results lack the ability to find a significant biomarker for males at risk for thrombotic events during COVID-19, which may be due to sample size.

Other studies have also suggested that age plays a role in thrombosis in COVID-19. Ages 55 years or older tend to be a significant risk factor for a venous thromboembolic event with COVID-19 [50]. Advanced age is also associated with a higher risk of mortality in patients with COVID-19 [51]. Our findings of the predictive capacity of citH3 and neutrophil count in patients younger than 60 could indicate screening biomarkers for patients in this population.

These results may also be relevant to other infections and may inform potential mechanisms for treatment development for other respiratory diseases with thrombotic complications.

Looking forward, these results may be applicable to patients hospitalized for severe respiratory conditions, not only in those with SARS-CoV-2 infection. In future studies, age, sex, race, and severity-matched non-COVID-19 patients should be assessed to understand if our findings are COVID-19-specific or generalizable for many upper respiratory conditions.

## 5. Limitations

There are several limitations to this study. First, the time of onset of COVID-19 symptoms and the type of treatment each patient received, especially prior to their admission at our hospital, differed between patients. The average time from symptom onset for non-thrombotic event patients was 7.7 days (range 1–17). For thrombotic event patients, their symptom-to-admission rate was 8.29 days (range 1–30). These factors could influence the plasma biomarkers. Along with the lack of clarity surrounding infection date and pre-hospital medical management, the method by which the blood samples were collected and processed must also be examined for limitations. The blood samples were stored on ice immediately after collection and prepared cold throughout the processing protocol. While this protocol has been internally validated with respect to plasma biomarkers in studies not related to thrombosis, this process may promote the activation of platelets and neutrophils. One study showed that vWF is 9.4% lower in blood stored at ice for 4 h compared to the same blood stored at room temperature [52]. Another study showed that long-term blood storage on ice induced neutrophil activation that was significantly elevated by 6 h, suggesting that for the most accurate results, the neutrophil phenotype should be assessed within 3 h of the blood draw [53]. Our protocol requires the blood to be processed within a maximum time frame of 3 h after sample collection, and this time frame will not have a clinically significant effect on the markers analyzed in our study. In addition, all the samples were exposed to the same protocol, so any slight activation within the markers would be nullified, as the percentage of change among all the samples would be the same. In addition, differing types of thrombotic events, such as stroke and deep vein thromboses, may influence biomarker levels in varying mechanisms. Although we found that citH3 is a significant risk factor in predicting a thrombotic event across all patients, and this is one of the largest studies of these biomarkers in COVID-19, the sample size was small, and certain groups (i.e., Asians) were underrepresented in our cohort.

With the addition of more samples, a significant difference in severity levels may become more apparent. With our current sample size, there was a significant difference in severity levels only when grouped into broader classification groupings.

## 6. Conclusions

Our findings suggest that NETs may be involved in the thrombotic complications of COVID-19, and these thrombotic events contribute to multi-system organ failure and mortality in COVID-19. In addition, citH3 may serve as a reliable biomarker for measuring the future risk of thrombotic events in COVID-19 patients. Even with the advances in vaccines, these complications are still very relevant. Understanding the biological underpinnings of this disease and its thrombotic complications can improve therapeutic interventions to reduce the burden of thrombosis in patients with COVID-19. This study emphasizes the importance of understanding NET markers and vWF levels of COVID-19 patients with thrombotic events in order to better inform the prevention of future thrombotic events due to coagulopathy in COVID-19 and other related illnesses.

## Figures and Tables

**Table 1:** Baseline characteristics.

Characteristics		Overall N = 156	No Thrombotic Event N = 100	Thrombotic Event N = 56	*p*-Value
Age, years, median [IQR]		58.00 [48.00, 68.00]	58.00 [49.00, 68.25]	55.50 [46.00, 65.50]	0.281
Sex, N (%)					
	Female	63 (40.4)	42 (42.0)	21 (37.5)	0.704
	Male	93 (59.6)	58 (58.0)	35 (62.5)	
Race, N (%)					
	Black	40 (25.6)	24 (24.0)	16 (28.6)	0.719
	White	79 (50.6)	53 (53.0)	26 (46.4)	
	Other	37 (23.7)	23 (23.0)	14 (25.0)	
COVID-19 severity, N (%)					
	Non-critical	95 (60.9)	76 (76.0)	19 (33.9)	<0.001
	Critical	61 (39.1)	24 (24.0)	37 (66.1)	
vWF, median [IQR]		23.37 [18.87, 28.22]	23.05 [18.61, 27.93]	24.01 [19.56, 29.64]	0.406
Citrullinated Histone, median [IQR]		40.78 [34.32, 51.73]	38.49 [31.60, 44.09]	46.65 [40.79, 61.03]	<0.001
Neutrophil Elastase, median [IQR]		349.87 [204.04, 635.42]	339.92 [190.36, 614.05]	433.16 [242.64, 733.19]	0.192
Myeloperoxidase, median [IQR]		3.86 [2.66, 5.18]	3.80 [2.64, 5.08]	4.10 [2.96, 5.53]	0.510
Segmented neutrophils, median [IQR]		82.10 [74.00, 87.88]	82.00 [74.20, 86.80]	83.40 [73.80, 89.35]	0.322
Neutrophil count, median [IQR]		7.50 [4.68, 11.03]	7.20 [4.40, 9.90]	8.30 [5.85, 13.65]	0.031

IQR, interquartile range. Characteristics of COVID-19 patients separated by thrombotic event and no-thrombotic event groups. There was a significantly decreased number of non-critical patients in the thrombotic event group. CitH3 was significantly elevated in the thrombotic event group. Neutrophil count was significantly elevated in the thrombotic event group.

**Table 2:** Result of univariable and multivariable logistic regression analysis to predict a thrombotic event in COVID-19 patients.

Effect	Univariable Analysis				Multivariable Analysis			
	OR	95% CIs		*p*-Value	Adjusted OR	95% Cis		*p*-Value
Age	0.98	0.96	1.01	0.1302	-			
Sex, Male vs. Female	1.21	0.62	2.36	0.5829	-			
Race, Black vs. Other	1.10	0.44	2.74	0.8459	-			
Race, White vs. Other	0.81	0.36	1.82	0.6031	-			
Severity	6.17	3.01	12.65	<0.0001	4.84	2.26	10.38	<0.0001
vWF	1.02	1.00	1.04	0.0844	0.96	0.91	1.02	0.1579
Citrullinated Histone	1.01	1.00	1.01	0.0159	1.02	1.00	1.04	0.0302
Neutrophil Elastase	1.00	1.00	1.00	0.6053	-			
Myeloperoxidase	1.07	0.97	1.18	0.2084	-			
Segmented neutrophil	1.00	0.97	1.03	0.9310	-			
Neutrophil count	1.08	1.01	1.15	0.0257	1.08	1.00	1.16	0.0508

OR, odds ratio, CI, confidence intervals. Univariable and multivariable analyses were performed to determine whether any of the demographic variables or biomarkers had the capacity to predict a thrombotic event in COVID-19 patients. Severity, citH3, and neutrophil count were shown to be significant predictors in both univariable and multivariable analysis.

**Table 3:** Result of multivariable logistic regression analysis to predict a thrombotic event in COVID-19 patients by race, age, and sex.

Sub-Cohort	Effect	Adjusted OR	95% CIs		*p*-Value
Black					
	Severity	5.08	1.18	21.92	0.0295
	vWF	0.99	0.89	1.10	0.8300
	Citrullinated Histone	1.00	0.98	1.03	0.8686
	Neutrophil count	1.05	0.91	1.21	0.4818
White					
	Severity	6.40	1.79	22.86	0.0042
	vWF	0.90	0.81	1.00	0.0505
	Citrullinated Histone	1.05	1.01	1.08	0.0105
	Neutrophil count	1.18	1.02	1.37	0.0277
Other					
	Severity	7.53	1.33	42.58	0.0224
	vWF	1.05	0.77	1.42	0.7691
	Citrullinated Histone	1.03	0.94	1.12	0.5145
	Neutrophil count	0.94	0.80	1.10	0.4343
Male					
	Severity	5.00	1.88	13.28	0.0012
	vWF	0.98	0.92	1.05	0.6449
	Citrullinated Histone	1.01	0.99	1.03	0.2181
	Neutrophil count	1.05	0.96	1.16	0.3169
Female					
	Severity	6.87	1.73	27.33	0.0062
	vWF	0.84	0.72	0.98	0.0305
	Citrullinated Histone	1.05	1.01	1.09	0.0206
	Neutrophil count	1.09	0.96	1.23	0.1793
Age < 60					
	Severity	3.01	1.00	9.60	0.0500
	vWF	0.91	0.91	0.80	0.1889
	Citrullinated Histone	1.05	1.05	1.00	0.0343
	Neutrophil count	1.15	1.15	1.02	0.0271
Age ≥ 60					
	Severity	7.65	2.32	25.26	0.0008
	vWF	0.99	0.92	1.05	0.6482
	Citrullinated Histone	1.01	0.99	1.03	0.4129
	Neutrophil count	1.02	0.92	1.13	0.7702

OR, odds ratio, CI, confidence intervals. A multivariable logistic regression analysis was used to analyze certain predictors in race, sex, and age categories to determine if these predictors were still significant in predicting a thrombotic event in COVID-19 patients. Severity was shown to still be a significant predictor across all categories, regardless of race, age, or sex. VWF was shown to be a significant predictor in the White cohort and the female cohort. CitH3 remained a significant predictor in the White cohort, the female cohort, and the age less than 60 cohort. Neutrophil count remained a significant predictor in the White cohort and the age less than 60 cohort.

**Table 4:**
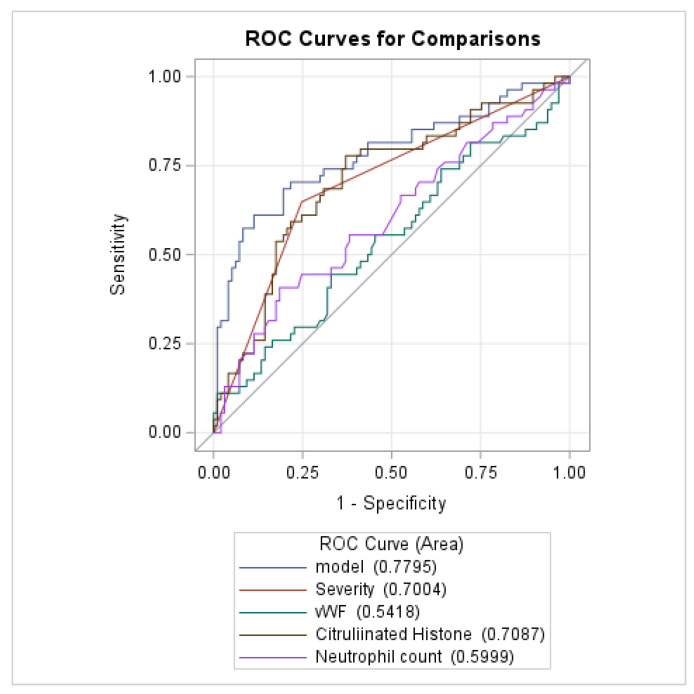
Comparison of Area under the ROC curve (AUC) with predictors for distinguishing patients with and without thrombotic events in COVID-19 patients.

ROC Model	AUC	Standard Errors	95% Wald CIs		*p*-Value
Model	0.7795	0.0425	0.6962	0.8628	<0.0001
Severity	0.7004	0.0395	0.6229	0.7778	<0.0001
vWF	0.5418	0.0504	0.4431	0.6405	0.4066
Citrullinated Histone	0.7087	0.0451	0.6203	0.7971	<0.0001
Neutrophil count	0.5999	0.0494	0.5031	0.6968	0.0432

ROC, Receiver Operating characteristic Curve. Model = Severity + vWF + Citrullinated Histone + Neutrophil count. An area under the ROC curve analysis was performed to model the significant predictors for thrombotic events in COVID-19. When combining severity, vWF, citH3, and neutrophil count into one model, a strong capacity to predict thrombotic events was shown, confirming our previous results. This was also demonstrated when using models of severity, citH3, and neutrophil count on their own, with citH3 having the strongest independent predictive capacity, as demonstrated by its higher AUC.

**Table 5:** Spearman’s Rho correlation in COVID-19 patients.

**(A) COVID-19 Patients with Thrombotic Events**						
		**Citrullinated Histone**	**Neutrophil Elastase**	**Myeloperoxidase**	**Segmented Neutrophils**	**Neutrophil Count**
vWF	ρ	0.75	−0.16	0.06	−0.20	−0.16
	*p*	<0.0001	0.2579	0.647	0.1434	0.2518
	N	55	53	55	54	54
Citrullinated Histone	ρ	-	−0.16	0.15	0.03	−0.03
	*p*		0.2349	0.2695	0.8233	0.802
	N		54	56	55	55
Neutrophil Elastase	ρ	-	-	0.13	0.22	0.11
	*p*			0.3594	0.1124	0.4081
	N			54	54	54
Myeloperoxidase	ρ	-	-	-	0.13	0.10
	*p*				0.339	0.4713
	N				55	55
Segmented neutrophils	ρ	-	-	-	-	0.59
**(B) COVID-19 Patients Without Thrombotic Events**						
		**Citrullinated Histone**	**Neutrophil Elastase**	**Myeloperoxidase**	**Segmented Neutrophils**	**Neutrophil Count**
vWF	ρ	0.67	0.03	−0.03	−0.08	−0.29
	*p*	<0.0001	0.7515	0.7673	0.4257	0.0038
	N	100	99	98	97	97
Citrullinated Histone	ρ	-	0.14	0.03	−0.08	−0.22
	*p*		0.1823	0.752	0.4256	0.0269
	N		99	98	97	97
Neutrophil Elastase	ρ	-	-	0.32	0.30	0.29
	*p*			0.0016	0.003	0.0037
	N			97	96	96
Myeloperoxidase	ρ	-	-	-	0.14	0.24
	*p*				0.1733	0.0217
	N				95	95
Segmented neutrophils	ρ	-	-	-	-	0.57
	*p*					<0.0001
	N					97

A Spearman’s rho correlation was performed in both the thrombotic event (A) and the no-thrombotic event (B) cohorts to see if any of the biomarkers and coagulation markers were significantly correlated. In the thrombotic event group, citH3 and vWF were significantly correlated and neutrophil count and segmented neutrophils were significantly correlated. In the no-thrombotic event group, vWF was significantly correlated with citH3 and with neutrophil count. CitH3 and neutrophil count were also significantly correlated. Neutrophil elastase was significantly correlated with myeloperoxidase, segmented neutrophils, and neutrophil count. Myeloperoxidase was significantly correlated with neutrophil count, and segmented neutrophils were significantly correlated with neutrophil count.

## Data Availability

Data will be made available on request.
